# Dissection de l'aorte thoracique descendante et de l'aorte abdominale dans la maladie de Takayasu: à propos d'un cas

**DOI:** 10.11604/pamj.2014.17.196.2052

**Published:** 2014-03-13

**Authors:** Abdelaziz Zaghdoudi, Monika Bukta, Mohamed Ali Mongalgi, Kais Malouche, Sonia Malouche

**Affiliations:** 1 Hôpital Mahmoud El Matri de l'Ariana, Rue Ibn Khaldoun, Ariana, Tunisie; 2 Institut Hedi Rais d'Ophtalmologie de Tunis, Boulevard 9 Avril, Bab Saadoun, Tunis, Tunisie; 3 Hopital Mongi Slim de La Marsa, Sidi Daoud, La Marsa, Tunisie

**Keywords:** Maladie de Takayasu, dissection aortique, angioscanner, diagnostique différenciel, âge, maladie de Horton, Takayasu disease, aortic dissection, CT angiography, differential diagnosis, age, Horton disease

## Abstract

La dissection aortique est une complication très rare de la maladie de Takayasu. Dans la littérature récente seulement 14 cas ont été publiés. Nous rapportons le cas d'un maghrébin âgé de 67 ans, présentant une dissection aigue de l'aorte thoracique descendante et de l'aorte abdominale avec un syndrome inflammatoire, révélateur d'une maladie de Takayasu. Le type de l'atteinte aortique (type III b, selon la classification de De Bakey et Standford) confirmé par angioscanner chez notre patient, n'a pas été rapporté. Il nous parait que l'artérite de Takayasu est sous-estimée chez les malades dépassant l’âge de la 4ème décennie. La forme tardive de cette pathologie pose un problème de diagnostic différentiel avec la maladie de Horton, selon les critères d’âge et d'ethnie. Actuellement la majorité des auteurs donnent la priorité à l'imagerie médicale, avec ou sans syndrome inflammatoire pour la confirmation du diagnostic de la maladie de Takayasu.

## Introduction

La maladie de Takayasu (MT) est une artérite très rare, d'origine inconnue, décrite pour la première fois au Japon en 1908 par MikitoTakayasu. Il s'agit d'une atteinte des vaisseaux de gros et de moyen calibre, touchant essentiellement l'aorte et ses principales branches. La dissection aortique est une complication exceptionnelle de cette maladie. Durant plus d'un siècle la MT n'a cessé d'intéresser les praticiens et les chercheurs dans les 5 continents. Le diagnostic de la MT représente un vrai défi pour les cliniciens malgré les critères diagnostiques de classification de l'American College of Rheumathology (Table 1)[1] et d'Ishikawa modifiés par Sharma (Table 2) [2]. Cette pathologie est considérée comme une affection propre à la jeune femme, mais elle se voit à toute tranche d’âge. Des cas de pédiatrie ont été rapportés ainsi que des cas après la 4eme décennie [3, 4].

**Table 1:** Critères de classification de l'American College Of Rheumatology [1]

Age de début ≤ 40 ans
Claudication des membres
Diminution d'au moins un pouls brachial
Différence de pression artérielle systolique ≥ 10mmHg entre les deux bras
Souffle sur une artère sous-clavière ou l'aorte abdominale
Anomalie artériographique : sténose ou occlusion de l'aorte ou de ses branches ou des artères
Proximales des membres supérieurs ou inférieurs, non liés à l'athérosclérose ou à une dysplasie fibromusculaire
Le diagnostic d'artérite de Takayasu est retenu sur trois critères ou plus des six ci-dessus, avec une sensibilité de 90,5% et une spécificité de 97,8%.
[1]. Arend WP, Michel BA, Bloch DA, Hunder GG, Calabrese LH, Edworthy SM, et al. The American College of Rheumathology 1990 criteria for the classification of Takayasu's arteritis. Arthritis Rheum. 1990; 33(8): 1129-34.

**Table 2:** Critères diagnostiques d'Ishikawa modifies par Sharma et al. [2]

**Critères majeurs**
Sténose ou occlusion de la portion moyenne de l'artère sous-clavière gauche en artériographie.
Sténose ou occlusion de la portion moyenne de l'artère sous-clavière droite en artériographie.
Symptômes caractéristiques d'une durée >1 mois : claudication, abolition d'un pouls ou anisotension, fièvre, carotidodynies, amaurose, troubles visuels, syncope, dyspnée, palpitations
**Critères mineurs**
Vitesse de sédimentation > 20 mm/h
Carotidodynies
Pression artérielle (PA) brachiale >140/90 mmHg ou PA poplitée > 160/90 mmHg
Insuffisance aortique ou dilatation de l'anneau aortique
Lésion des artères pulmonaires
Sténoses ou occlusion de la portion moyenne de la carotide gauche en artériographie
Sténose ou occlusion du tiers distal du tronc brachiocéphalique en artériographie
Lésion de l'aorte thoracique descendante en artériographie
Lésion de l'aorte abdominale en artériographie
Lésion coronarienne avec âge < 30 ans en absence de dyslipidémie ou diabète
La présence de deux critères majeurs ou d'un critère majeur plus deux critères mineurs ou de quatre critères mineurs permet de retenir le diagnostic de la maladie de Takayasu avec une sensibilité de 92,5% et une spécificité de 95%.
[2] Sharma BK, Jain S, Suri S, Numano F. Diagnostic criteria for Takayasu arteritis. Int J Cardiol 1996; (Suppl.54): S141-7

## Patient et observation

Mr.S.A, maghrébin, âgé de 67 ans, sans antécédents pathologiques notables, consultait pour vomissement, diarrhée et douleur abdominale diffuse accentuée dans la région épigastrique. L'examen clinique trouvait un patient apyrétique, un abdomen souple, une douleur épigastrique à la palpation. L'examen cardiovasculaire était strictement normal: la tension artérielle était 120/80 mmHg des deux côtés, pas de souffle cardiaque, ni carotidien, ni au niveau de l'aorte abdominale. Les pouls périphériques étaient présents et symétriques. Le reste de l'examen clinique: pulmonaire, neurologique, osteoarticulaire et dermatologique était sans particularité. Le bilan biologique: numération formule sanguine (NFS), vitesse de sédimentation (VS), glycémie, urée, ionogramme, transaminases, amylasémie et troponine était normal. L'examen radiologique: la radiologie de thorax, l'abdomen sans préparation, l’échographie abdomino-pelvienne ne montrait aucune anomalie. Au bout de 12 heures l’état clinique du patient s'améliorait. Le diagnostic probable était une gastroentérite aigue.

Une semaine après le patient était hospitalisé pour une altération de l’état général, marquée par une épigastralgie, une douleur abdominale atroce et une fébricule. Un bilan biologique en urgence notait une VS à 60 mm/H, une C-réactive protéine(CRP) à 158 mg/l et une fibrinémie à 7.2 g/dl. Une fibroscopie digestive trouvait une hernie hiatale et une antrobulbite congestive, qui n'expliquaient pas cette douleur.

**Figure 1:**
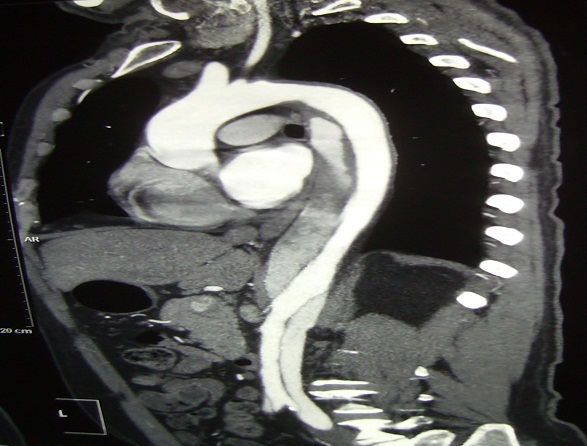
Angioscanner thoraco-abdominal montrant la dissection de l'aorte thoracique descendante débutant au niveau de l'isthme aortique et s’étendant jusqu’à l'aorte abdominale et les artères iliaques primitives ainsi que externes droite et gauche

**Figure 2:**
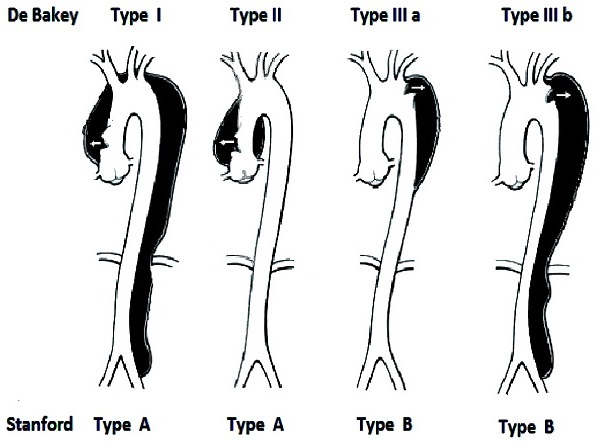
Classification des dissections aortiques selon De Bakey et Stanford

L'angioscanner thoraco-abdominale en urgence montrait une dissection de 33 cm de l'aorte thoracique descendante et de l'aorte abdominale jusqu’à la bifurcation des artères iliaques (Figure 1). C’était une dissection aortique aigue stade III b selon la classification de De Bakey et de Stanford (Figure 2).

Le patient était transféré en réanimation cardiovasculaire. Une intervention chirurgicale en urgence était écartée vu la localisation de la dissection et l'absence des manifestations ischémiques. Le diagnostic de la Maladie de Takayasu était évoqué dans ce contexte inflammatoire. Une recherche d’étiologie de cette dissection aortique était entreprise. Le bilan infectieux: tuberculose, syphilis était négatif, une autre maladie inflammatoire dite systémique: lupus, Behcet, polyarthrite, sarcoïdose, Wegener, fibrose retropéritoniale, maladie de Cogan, était peu probable. Vu l’âge de notre patient une biopsie unilatérale de l'artère temporale était pratiquée, l'examen anatomopathologique ne montrait aucun signe en faveur de la maladie de Horton. L'examen ophtalmologique était normal.

L’écho-Doppler artériel du tronc supraaortique trouvait un discret athérome diffus circonférentiel essentiellement au niveau des bifurcations carotidiennes sans sténose hemodynamiquement significatif. Le diagnostic de la MT était retenu. Un traitement à base de prednisone à la dose de 0.5 mg/k/j était efficace. Trois semaines plus tard la CRP était à 10.9 mg/l, la VS à 27 mm/h. L’évolution clinique était favorable, la température revenait à la normale, avec disparition de la douleur, l'angioscanner de contrôle montrait la même image radiologique.

La décroissance progressive de la corticothérapie était entamée après un mois de traitement d'attaque, avec surveillance biologique et radiologique. Une année plus tard une corticothérapie de 10 mg de prednisone était maintenue, avec un béta bloquant pour optimaliser la tension artérielle. La CRP et la VS gardaient une valeur normale. Le 4ème angioscanner montrait une thrombose partielle du faux chenal au niveau de la région thoracique avec une légère dilatation de l'aorte dans ce territoire. L’état général du patient était satisfaisant.

## Discussion

La MT est une artérite de cause encore inconnue, affectant l'aorte, ses principales branches et les artères pulmonaires. L'atteinte aortique concerne environ 60% des patients, mais les dissections de l'aorte sont des complications extrêmement rares [5]. L’épidémiologie de cette maladie et les lésions préférentielles des vaisseaux selon les régions géographiques et selon l’âge des patients est loin d’être éclairée.

Les constatations actuelles trouvent que la MT atteint préférentiellement les patients originaires d'Asie, d'Afrique ou d'Amérique du sud. L'incidence varie entre 1.2 et 3.6 cas par an et par million d'habitant [6]. Cette pathologie touche les femmes avant la 4eme décennie dans près de 90% des cas [7]. Plusieurs études confirment que la MT n'est pas exceptionnelle après la quarantaine. La série monocentrique de L. Arnaud et al. confirme que 25% des patients ont dépassé l’âge de la quarantaine [4]. Les formes tardives sont observées essentiellement chez les caucasiens. Les formes pédiatriques ne sont pas exceptionnelles [3].

L’étude anatomo-pathologique de l'artérite de Takayasu montre des vaisseaux épaissis et fibreux, qui peuvent être le siège de sténose ou anévrisme. L'examen microscopique trouve une panartérite marquée par l'infiltration lymphocytaire et macrophagique au niveau de l'adventice et du média de la paroi artérielle. Contrairement à la maladie de Horton, la limitante élastique interne est généralement respectée. L'atteinte inflammatoire des vaisseaux s'explique par une pathologie dysimmunitaire [8].

Actuellement ni les biopsies artérielles, ni la preuve histologique ne sont pas indispensables au diagnostic de la maladie de Takayasu [4]. Généralement la MT évolue en deux phases caractérisées par des manifestations cliniques: la phase préocclusive, qui peut être absente ou discrète. Si les signes existent, ils se manifestent sous forme d'altération de l’état général, de myalgie, d'arthralgie, fièvre, sueurs nocturne d’épanchement pleural, de péricardite, d’épisclérite, d’érythème noueux [9]; la phase occlusive, qui peut être d'emblée la phase unique de cette maladie, sous forme de manifestation vasculaire (ischémique, sténosante, anévrismale, disséquant) avec ou sans syndrome inflammatoire [4].

La symptomatologie de la première phase peut disparaitre après quelques semaines, comme elle peut rester latente durant 5 ans en moyenne avant l'apparition de la phase occlusive. Le cas de notre patient maghrébin débutait par la phase occlusive dans le contexte d'un syndrome inflammatoire. L'angioscanner était un élément de diagnostic, montrant une dissection de l'aorte thoracique descendante et de l'aorte abdominale jusqu’à la bifurcation des deux artères iliaques. Au cours de recherche étiologique une pathologie infectieuse et inflammatoire systémique étaient éliminées. Par contre cette dissection aortique peut entrer dans le cadre de la maladie de Horton, avec l'atteinte des vaisseaux de gros calibre, le syndrome inflammatoire (VS, CRP), l’âge et l’évolution favorable sous corticothérapie. Devant l'absence des signes céphaliques: céphalée temporopariétale, claudication des mâchoires, la négativité de la biopsie temporale et de l'atteinte oculaire, la maladie de Horton était peu probable. Le diagnostic de la MT était retenu.

Dans la métaanalyse de T.Kalife et col. 14 cas de MT avec une dissection aortique ont été recensés dans la littérature. Parmi ces 14 cas on trouve 4 cas de dissection abdominale, 6 cas de dissection de l'aorte ascendante et 2 dissections thoracique [5]. Notre cas n'a pas été rapporté dans la littérature.

La rareté de la MT explique le manque des essais contrôlés du traitement médical. Lorsque la maladie parait initialement active, la majorité des équipes préconise un traitement d'attaque de 0.7 à 1 mg/kg/j. Dans notre cas la dose initiale était de 0.5mg/kg/j durant un mois. Après une régression progressive jusqu’à 10 mg/j était entreprise pendant 10 mois. Cette dose était suffisante pour la stabilité clinique et biologique du patient. Dans la majorité des séries, le traitement par corticothérapie ne permet pas une rémission que dans 50% des cas, d'où la nécessité du traitement par immunosuppresseur [9, 10].

La chirurgie vasculaire de la dissection aortique type B est complexe et grevée d'une lourde mortalité ou de séquelles paraplégiques. Ce genre d'intervention doit être effectué par une équipe très expérimentée.

## Conclusion

Nous rapportons le cas d'une dissection aortique, chez un maghrébin âgé de 67 ans, révélatrice de la MT. Il s'agit d'une atteinte très rare, grave qui nécessite une équipe multidisciplinaire. L'angioscanner est incontournable pour le diagnostic et le suivi de la maladie. Malgré les critères de la classification, le diagnostic différenciel avec la maladie de Horton reste un vrai défi pour les praticiens. Seule une meilleure compréhension de la physiopathologie de la MT pourra nous orienter vers le diagnostic positif.
